# Spinal cord and cutaneous involvement in paracoccidioidomycosis

**DOI:** 10.1590/0037-8682-0115-2021

**Published:** 2021-04-28

**Authors:** Andrea Fernandes Eloy da Costa França, Paulo Eduardo Neves Ferreira Velho, Fabiano Reis

**Affiliations:** 1 Universidade Estadual de Campinas, Faculdade de Ciências Médicas, Departamento de Clínica Médica, Disciplina de Dermatologia, Campinas, SP, Brasil.; 2 Universidade Estadual de Campinas, Faculdade de Ciências Médicas, Departamento de Radiologia, Campinas, SP, Brasil.

**FIGURE 1:**
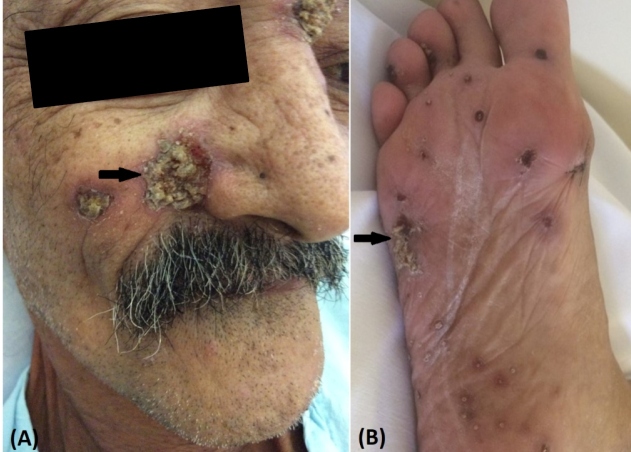
Skin ulcers with yellow crusts on the face (arrow) **(A)**. Necrotic ulcers on soles (arrow) **(B)**.

**FIGURE 2:**
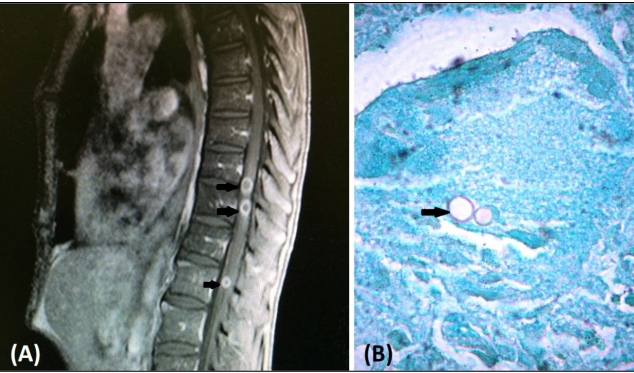
Sagittal T1 weighted images after contrast administration demonstrate intramedullary nodular enhancement at thoracic and lumbar levels (arrows) **(A)**. Broad-based yeast cells (arrow) at histological samples **(B)**.

A 62-year-old man, a gardener, was hospitalized in Southeast Brazil with a four-month history of weight loss, progressive lower limb paresthesia, muscle weakness, urinary and bowel incontinence, and skin lesions. On clinical examination, he had paraplegia and presented skin ulcers measuring two centimeters on the face and soles (Figure 1). Magnetic resonance imaging showed intramedullary nodular lesions at the thoracic (T10 -T11) and lumbar levels (L1) with peripheral enhancement (Figure 2A). 

Identification of broad-based budding yeast cells on potassium hydroxide examination of cutaneous imprints confirmed paracoccidioidomycosis, which was also found in the microscopic analysis of a skin biopsy (Figure 2B). The patient was treated with amphotericin B deoxycholate during the in-hospital stay, and then released after being prescribed trimethoprim/sulfamethoxazole.

Paracoccidioidomycosis is a systemic mycosis endemic in Brazil, caused by the dimorphic fungus *P. brasiliensis*. Infection occurs by inhalation of conidia from the soil. The prevalence of neurological manifestations varies from 9% to 25%. The central nervous system is affected by the hematological or lymphatic spreading of the fungus. Cerebral hemispheres are the most common site of neurological paracoccidioidomycosis, but clinical presentation depends on the location of the lesions^1^ ^,^ ^2^. Spinal cord involvement is rare (4% of cases) and represents 0.6% of all manifestations of paracoccidioidomycosis^3^. The fungus is unlikely to be found in the cerebrospinal fluid, and a biopsy may be difficult to access depending on the neurological site. In this scenario, the clinical suspicion of paracoccidioidomycosis based on skin lesions allowed an early diagnosis, avoiding permanent sequelae.
